# The role of the endogenous oxytocin system under psychosocial stress conditions in adolescents suffering from anxiety disorder: study protocol for a parallel group controlled trial

**DOI:** 10.1186/s40359-021-00564-z

**Published:** 2021-04-26

**Authors:** Leonie Goetz, Irina Jarvers, Daniel Schleicher, Kathrin Mikan, Romuald Brunner, Stephanie Kandsperger

**Affiliations:** Clinic for Child and Adolescent Psychiatry, Psychosomatics and Psychotherapy, University of Regensburg, Universitätsstraße 84, 93053 Regensburg, Germany

**Keywords:** Anxiety disorder, Case–control study, Oxytocin, Cortisol, Trier social stress test, Stress mechanisms, Children and adolescents

## Abstract

**Background:**

In social neuroscience, the linkage between the endocrinological system and the etiology and symptomatology of mental health problems has received increasing attention. A particular focus is given to the neuropeptide oxytocin with its anxiolytic and stress-buffering effect and the resulting therapeutic potential for anxiety disorders. Even though anxiety disorders are the most prevalent mental health disorders in childhood and adolescence worldwide, the reactivity of the endogenous oxytocin system to an acute stressor (Trier Social Stress Test, TSST) has so far only been investigated in healthy children. It has been shown that peripheral oxytocin levels increased under psychosocial stress conditions. In the present study, it is hypothesized that the endogenous oxytocin system in children and adolescents suffering from a clinically diagnosed anxiety disorder is dysregulated. Three primary outcome parameters will be analyzed: significant differences between participants with anxiety disorders compared to healthy controls in basal oxytocin levels, varying salivary oxytocin release after stress exposure and the correlation between the cortisol peak/-decrease and oxytocin level over time. Secondary outcome criteria are significant differences in physiological (heart rate) and psychological (perceived stress, anxiety, insecurity, tension) responses.

**Methods:**

The present study is a single-center experimental observation study to investigate the reactivity of the endocrinological system to a psychosocial stressor (TSST). 32 children and adolescents (11–18 years) suffering from anxiety disorder will be compared to a matched healthy control group. After a detailed psychological assessment, saliva samples will be taken to measure oxytocin levels before and after psychosocial stress exposure at eight different time points. Additionally, the stress hormone cortisol will be analyzed according to the same procedure.

**Discussion:**

Due to the high prevalence and comorbidity rate with numerous other psychiatric disorders and mental health problems, there is an urgent need to strengthen research in possible neurobiological underpinnings of anxiety disorders. To our knowledge, the proposed experiment is the first study to examine the endocrinological oxytocin and cortisol reaction to an acute psychosocial stressor in children and adolescents with mental health disorders.

*Trial registration* The study is registered in the German Clinical Trials Register since 11 September 2019, DRKS00017793, https://www.drks.de/drks_web/navigate.do?navigationId=trial.HTML&TRIAL_ID=DRKS00017793.

**Supplementary Information:**

The online version contains supplementary material available at 10.1186/s40359-021-00564-z.

## Background

Anxiety disorders are the most prevalent mental health disorders in childhood and adolescence [1, 2]. The Mental Health Module (BELLA study) of the National Health Interview and Examination Survey for Children and Adolescents (KiGGS, Germany) suggests that 1 out of 10 children and adolescents aged 7–17 shows mental health problems associated with high levels of anxiety [3]. Anxiety disorders can lead to mental health problems along with adverse physiological functioning and impairments in adulthood [4, 5] and have high comorbidity rates with numerous other psychiatric disorders such as depressive disorders (comorbidity rate ranges from 50 to 72%) [5]. Whereas anxiety disorders such as phobias, social anxiety and separation anxiety have a very early age of onset between 5 and 10 years, others (generalized anxiety disorder, panic disorder) tend to have a later age of onset between 24 and 50 years [6]. Due to the high number of younger patients, it is an essential task for early prevention to investigate the etiopathology of anxiety disorders, particularly in childhood and adolescence.

Of particular interest is the relevance of endocrinological functions to the onset and course of mental diseases. With a better understanding of the endocrinological system and its effects on social behavior, it may be possible to develop new drug therapy strategies in addition to psychotherapeutic approaches. According to the guidelines of the German Society for Child and Adolescent Psychiatry, Psychosomatics and Psychotherapy (DGKJP), classical psychotropic drugs such as tricyclic antidepressants and benzodiazepines do not show adequate effects in children on panic disorders, and neither do tricyclics on separation anxiety disorders [7]. Tricyclics are less preferred in the treatment of anxiety in children due to their potential for cardiac abnormalities [8] and benzodiazepines because of their marked side effects such as the development of addiction [9]. Merely the efficacy of Selective Serotonin Reuptake Inhibitors (SSRIs) on generalized anxiety disorders and separation anxiety disorders in childhood and adolescence has been confirmed with limitations (for review see [10–12]).

In clinical neuroscience, worldwide the neuropeptide oxytocin (OXT) has started to receive increasing attention for its role in mental health and stress regulation [13–16]. Several recent studies with healthy humans and adult patients with social anxiety disorder describe a reduction of experienced anxiety and a stress-buffering effect of intranasal (i.n.) OXT (e.g. [14, 17]).

OXT is primarily synthesized in magnocellular neurons in the paraventricular and supraoptic nuclei of the hypothalamus and released into the systemic circulation by the posterior pituitary [17]. OXT acts as a neurohormone as well as a neuromodulator in many OXT-Receptors (OXTR) expressing brain regions such as the hypothalamic nuclei and extrahypothalamic limbic areas [18]. In addition to the central OXT and OXTR expression, OXTR and OXT synthesis have also been detected in peripheral organs [14, 19]. The OXT concentration can be quantified in the cerebrospinal fluid as well as in blood plasma, urine and saliva samples representing either the cerebral or peripheral OXT system [20]. Since fearful and stressful conditions activate the endogenous OXT system and consequently, central as well as peripheral OXT is being released, peripheral saliva OXT can be assessed as a global marker of the OXT system and to some extent represents the central activity of the individual OXT system to stress [15].

Numerous studies in animals and healthy adult volunteers have demonstrated an anxiolytic and prosocial effect of OXT (for review see [15, 16]). OXT seems to play an important role in human social cognition, motivation, emotion detection, emotion recognition [21] and emotional memory as well as prosociality such as trusting behavior, generosity and cooperation [22–26]. OXT modulates the salience processing of external social cues constrained by personality characteristics and different contextual factors [23, 25]. Human neuroimaging studies have demonstrated altered amygdala reactivity reflecting different i.n. induced OXT activity in nodes of the salience network [25]. Compared to placebo for example, after i.n. OXT administration, participants with post-traumatic stress disorder (PTSD) and borderline personality disorder showed decreased amygdala activity towards angry vs. happy faces. In contrast, healthy participants showed increased activity [25]. The individual and population-specific effects of exogenous i.n. OXT and modulational effects on social salience depending on individual factors might reflect the differences in the endogenous OXT response between various populations [23]. Neumann and Slattery [15] hypothesized that higher cerebral OXT activity is associated with less expression of fear. Patients who exhibited a current diagnosis of PTSD and participants with a history of childhood maltreatment had much higher peripheral blood OXT levels than healthy controls [13]. It was assumed that an acute release of peripheral and central OXT in stressful situations provides a natural mechanism for the reduction of stress and anxiety [27]. Lebowitz et al. [28] described that children with separation anxiety had a significant (*p* < 0.01) decrease in basal saliva OXT levels (16.7 pg/ml, SD = 706) compared to anxious children not meeting criteria for separation anxiety (26.8 pg/ml, SD = 15.6). The authors concluded that children with separation anxiety have greater reliance on the support of primary attachment figures which leads to an additional decrease in OXT level. It is assumed that attachment representations affect stress responses and positively predict OXT levels [29, 30]. Pierrehumbert et al. [30] noted that participants with insecure attachment showed elevated levels of subjective stress and moderate levels of OXT during the TSST. Participants with secure attachment reported relatively low subjective stress and presented high levels of OXT. Due to the correlation between OXT, attachment and stress response [29–31] as well as the evidence of other animal and human studies [15], it is hypothesized that the etiology of anxiety disorders, particularly those with a social component, may be associated with an imbalance of the endogenous OXT system.

Despite the high prevalence of anxiety disorders in adolescents (11–18 years), the potential therapeutic use and anxiolytic effect of OXT (see above), as well as the influence of stress on the endogenous OXT system of children and adolescents, have only been investigated in one study with healthy volunteers by Bernhard et al. [32]. In a standardized laboratory setting the authors demonstrated that the peripheral OXT level increased under psychosocial stress conditions (using the TSST [33]). In addition, this study concluded that low basal OXT levels were associated with greater expression of anxiety and insecurity during stressful situations. Children and adolescents with a primary low basal OXT level showed a greater increase in OXT during the TSST. In contrast, a higher basal OXT level correlated subsequently with less anxiety [32]. Even though childhood experiences influence the regulation of OXT release in adulthood [13], there is no comparable trial investigating the response of the endogenous OXT system to an acute stressor in children and adolescents with psychiatric disorders, in particular, anxiety disorders compared to a control group of same-aged healthy participants without psychiatric diagnoses.

In the study by Bernhard et al. [32], the salivary cortisol (CORT) concentration was examined as a second neurophysiological parameter of stress reaction. The adrenal cortical hormone CORT is an elementary component of the hypothalamic–pituitary–adrenal (HPA) axis and the endocrinological stress regulation system. A meta-analysis by Cardoso et al. [34] supported the hypothesis that OXT plays an important role in the etiology of HPA dysfunction associated with psychopathology. The detailed interplay of OXT and CORT under stress conditions has rarely been studied [32]. In a human study in 2003, Heinrichs et al. [35] were the first to observe that the combination of i.n. OXT and social support suppressed CORT levels under stress conditions (TSST) and resulted in increased calmness and decreased anxiety. In healthy participants i.n. OXT, as well as natural high baseline OXT concentrations in saliva, appear to be correlated with low CORT response and less anxiety in stressful situations [32, 35, 36]. In contrast, lower baseline OXT was associated with higher CORT levels and greater OXT reactivity [32]. To the best of our knowledge, the only study addressing this relationship in children and adolescents was conducted by Bernhard et al. [32]. In the study, healthy children and adolescents showed an earlier peak of salivary OXT at + 1 min after stress compared to the CORT level at + 10 min after stress. Furthermore, they showed an earlier decrease of OXT to the baseline level compared to CORT after stress exposure was terminated [32]. Based on the earlier OXT peak, the authors assumed that OXT has a regulatory effect on CORT, which may act as a support mechanism during stressful situations [32]. In the present study, we expect a dysregulated interplay between OXT and CORT under stress [34] in children and adolescents suffering from anxiety disorder compared to healthy controls. If children and adolescents with anxiety disorder show a low basal OXT level (see above), there are two possible assumptions on the interplay between OXT and CORT. On the one hand, since low basal OXT levels in healthy participants correlated with a higher increase of saliva OXT and CORT during the TSST [32], we expect participants with anxiety disorder to show less increase of OXT, thereby reflecting a pathological lacking stress-buffering mechanism [15] and a subsequently higher expression of subjective stress and anxiety [35, 37]. On the other hand, because of the HPA axis co-activation and greater stress-induced OXT reactivity associated with faster vagal recovery, Engert et al. [38] rather suggested a “recovery-boosting” than a “reactivity-buffering” effect. With respect to previous research, however, the influence of OXT on the HPA axis under stress has not been fully determined yet, especially regarding clinical populations. Recent findings describe a blunted CORT response to psychosocial stress (TSST) in women with anxiety disorder [39] and patients with panic disorder [40, 41]. These findings led to the hypothesis of a HPA-axis hypo-responsiveness in patients with mental health disorders, promoting high vulnerability during acute stress response. This was also found in studies with adolescents with non-suicidal self-injury (NSSI) [42].

Due to the controversial findings [32, 35, 38], there is an urgent need for further research to improve our knowledge on the interplay of OXT and CORT under stress in psychiatric compared to healthy participants [35].

### Objectives

With the purpose of a better understanding of the linkage between dysregulation of the endogenous OXT system and the etiology of anxiety disorders, the present study aims to investigate three questions as main outcome objectives. First, it will be investigated whether basal saliva OXT levels of children and adolescents (11–18 years of age) with a clinically diagnosed anxiety disorder (experimental group) differ significantly from OXT levels of healthy children and adolescents without a clinical psychiatric diagnosis (healthy control group). The second question addresses whether children and adolescents of the experimental group show a significant change in the salivary OXT level to an acute stressor (TSST) compared to healthy controls. Regarding the key role of CORT in stress regulation, the third question addresses the differences of both groups between the in- and decrease of salivary CORT in relation to the OXT level under stress conditions. Secondary outcome criteria are significant differences in physiological (heart rate) and psychological (perceived stress, anxiety, insecurity, and tension) responses between both groups.

As an additional exploratory question, the severity of anxiety symptoms and the partial influence of comorbid psychiatric conditions such as depression will be evaluated.

### Primary hypothesis

It is hypothesized, that the endogenous OXT system of children and adolescents (11–18 years of age) suffering from a clinically diagnosed anxiety disorder is dysregulated. As different types of anxiety disorders in children have been associated with dysregulation of the HPA-axis [43], no differences between different types of anxiety disorders in response to stress are expected. A lower basal OXT level of the experimental group, as well as less OXT release as a response to an acute stressor, is expected, compared to the control group. In both groups, it is assumed that the OXT level peaks and decreases earlier than the CORT level. We expect a negative correlation between basal OXT level and CORT increase in both groups. Basal OXT and OXT increase under stress are expected to correlate positively with subjective stress level (VAS). OXT increase is expected to correlate positively with vagal recovery. In healthy controls, we anticipate a positive correlation between OXT release and CORT release. As described in the background section, in participants with anxiety disorders two controversial hypotheses about the interplay between CORT and OXT under stress will be discussed. First, participants with anxiety disorders might show low OXT increase correlating with high CORT increase reflecting a pathological, lacking stress-buffering effect of OXT. Second, participants with anxiety disorders might show low OXT increase correlating with attenuated CORT response reflecting HPA-axis hypo-responsiveness.

## Methods/design

### Design

The present trial is a single-center experimental observational study with a matched control group. An experimental group of children and adolescents with diagnosed anxiety disorder according to the criteria of the Diagnostic and Statistical Manual of Mental Disorders-IV (DSM-IV) and International Statistical Classification of Diseases and Related Health Problems-10 (ICD-10) will be compared to a matched control group of same-aged healthy children and adolescents. The procedure applies to both groups. Psychometric assessment, hormone assay, as well as physiological measurements (heart rate) will be investigated. The study protocol conforms to the Standard Protocol Items: Recommendations for interventional Trials (SPIRIT) checklist (See Additional file 1).

### Setting and recruitment

The study will be conducted at the Clinic for Child and Adolescent Psychiatry, Psychosomatics and Psychotherapy at the University of Regensburg, Germany. The aim is to include N = 64 children and adolescents aged 11–18 years. 32 patients with anxiety disorder and 32 healthy controls will be recruited from the region of Regensburg. Potential participants will be approached through the consecutive admission to the in- or outpatient clinic department, study flyers, via email distribution to colleagues and advertisements in the pediatric and psychiatric services. Upon showing interest in study participation, the potential participant will be contacted and initially screened for eligibility by telephone or personal conversation.

### Eligibility criteria

All participants and their legal guardians have to provide a sufficient understanding of the German language and a declaration of informed consent before taking part in the study. The study protocol has been approved by the ethical committee of the University of Regensburg. The experimental group will consist of children and adolescents meeting the criteria of an anxiety disorder according to the classification systems DSM-IV and ICD-10. Included diagnoses are Agoraphobia (ICD-10 F40.0) with/without panic disorder (F40.00, F40.01), social phobia (F40.1), panic disorder (F41.0), generalized anxiety disorder (F41.1), separation anxiety disorder of childhood (F93.0) as well as social (F93.2) and generalized (F93.80) anxiety disorder of childhood. The categorical diagnoses of diseases will be assessed using the semi-structured diagnostic interview M.I.N.I-KID 6.0 [44]. Anxiety-questionnaires (BAI [45], BDI-II [46], STAI [47], SPAIK [48], SCARED-D [49, 50]) will be administered to measure the symptomatology of anxiety dimensionally. Exclusion criteria will be IQ < 85 [assessed by the Wechsler Intelligence Scale-V (WISC-V), 4 subtests: Similarities, Vocabulary, Matrix Reasoning, Block Design; 11–16 years [51]; > 16 years [52]], pubertas praecox vera [53], pregnancy, current glucocorticoid-containing medication, known genetic syndromes, history of traumatic brain injury, history of endocrinological disorder, any other chronic neurological disorder which influences the brain development and physiology, acute suicidal behavior and any current and history of psychiatric disorder according to DSM-IV and ICD-10 except mild depressive episode (ICD-10 F32.0) and moderate depressive episode (F32.1). Patients with mild and moderate depressive episodes were included because of the high comorbidity rate between anxiety disorders and depression [5]. Hormonal contraceptives and psychotropic medication will be noted and corrected for but are no exclusion criteria. The healthy control group will be matched according to sex, age, pubertal status and school education. For the control group, exclusion criteria additionally include current and history of in- and outpatient psychiatric and psychotherapeutic treatments and any current or lifetime psychiatric disorders.

### Procedure

**Fig. 1:**
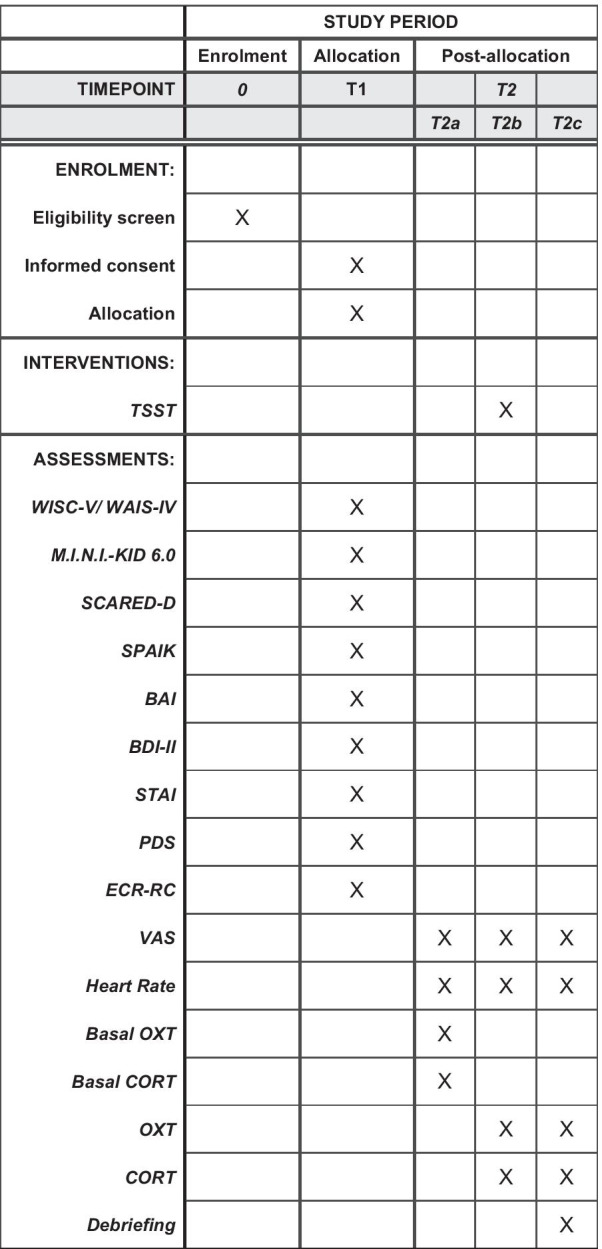
Schedule of Enrolment, Interventions and Assessments—Trial Procedure. *TSST* Trier Social Stress Test, *WISC-V* Wechsler Intelligence Scale for Children—V, *WAIS-IV* Wechsler Adult Intelligence Scale—Fourth Edition, *M.I.N.I-KID 6.0* Mini-international Neuropsychiatric Interview for children and adolescents, *SCARED-D* Screen for Child Anxiety Related Emotional Disorders—German Version, *SPAIK* Social Phobia and Anxiety Inventory for Children, *BAI* Beck Anxiety Inventory, *BDI-II* Beck Depression Inventory-II, *STAI* State-Trait Anxiety Inventory, *PDS* Pubertal Development Scale, *ECR-RC* Experiences in Close Relationships Scale—Revised Child version, *VAS* Visual Analogue Scale for perceived stress, anxiety, insecurity, tension, *OXT* Oxytocin, *CORT* Cortisol, *T2a* Time previous to TSST, *T2b* TSST, *T2c* Time after TSST

After recruitment, participants and their legal guardians will be provided with written information on background, aim, study procedure, risks and advantages of participation, data collection and privacy policy (T0, see Fig. 1). After obtaining informed and written consent by the children and adolescents and their legal guardians, participants will be screened for eligibility with semi-structured diagnostic interviews (see Psychological Measures) (T1, see Fig. 1). In a second appointment, the assessment of salivary hormone concentrations under stress conditions will take place (T2, see Fig. 1). Each appointment will last approximately 2.5 h. The TSST [33] (T2b, see Fig. 1) will be conducted in order to induce a psychological and physiological stress response in children (see Physiological Measures). See Fig. 1 for an overview of the trial procedure.

All children and adolescents will receive a 50 € gift voucher for participating. In case a participant and legal guardian withdraw their consent from participating at any given time, the data already obtained will be destroyed or only considered if written permission is obtained.

### Intervention

All participants will be exposed to an acute psychosocial laboratory stressor (TSST) while saliva samples will be taken. Participants with an anxiety disorder will receive in- and/or outpatient diagnostic/medical consultation/treatment (Treatment as usual, TAU) at the Clinic for Child and Adolescent Psychiatry, Psychosomatics and Psychotherapy, University of Regensburg. If the healthy controls show unexpected pathological psychiatric symptoms during the study procedure, they have access to detailed diagnostic/medical consultation/treatment options.

### Data assessment

#### Psychological measures

To determine eligibility criteria, semi-structured diagnostic interviews (see Table 1) will be conducted (T1, see Fig. 1). The German version of the Screen for Child Anxiety Related Emotional Disorders (SCARED-D) [50] will be used to screen the participants for anxiety disorders. The SCARED-D is a 41 item self-and parent-report questionnaire to identify children at the age of 9–18 in clinical samples while considering five different factors of main anxiety diagnoses (somatic/panic, general anxiety, separation anxiety, social phobia, school phobia) [49]. For detailed assessment of social anxiety and phobia according to the DSM-IV diagnostic criteria, the German Screening for Social Phobia and Anxiety Inventory for Children (SPAIK) [48] will be used. Here, 26 different situations representing psychological and physiological aspects of social phobia are presented to the child. The responder has to rate the frequency of occurrence of these situations on a three-point Likert-scale: 0 = never or hardly ever, 1 = sometimes, 2 = almost always or always [54]. The intensity of anxiety disorders according to the DSM-IV criteria will be assessed using the Beck Anxiety Inventory (BAI) [45]. Participants have to evaluate 21 statements according to how much they are bothered by the symptoms of anxiety experienced in the last seven days [55]. A total of 13 items assesses physical aspects of anxiety, five cognitive aspects and three relate to both.

**Table 1:** Applied measures

Measure	Duration min	Number of items	Timepoint	Information
SCARED-D	15	41	T1	Screen for Child Anxiety Related Emotional Disorders (SCARED)—German Version
SPAIK	15	26	T1	*Sozialphobie und -angstinventar für Kinder* (Social Phobia and Anxiety Inventory for Children)
BAI	10	21	T1	Beck Anxiety Inventory
BDI-II	10	21	T1	Beck Depression Inventory-II
STAI	10	40	T1	State-Trait Anxiety Inventory for adults (STAI)
M.I.N.I.- KID 6.0	45		T1	Mini-international Neuropsychiatric Interview for Children and Adolescents (M.I.N.I.-KID)
ECR-RC	10	12	T1	Short version of the Experiences in Close Relationships Scale—Revised Child version
PDS	5	6	T1	Pubertal Development Scale
VAS	2		T2	Visual Analogue Scale for perceived stress, anxiety, insecurity and tension
WISC-V/ WAIS-IV	20		T1	Wechsler Intelligence Scale for Children—V / Wechsler Adult Intelligence Scale—Fourth Edition; 4 subtests (Similarities, Vocabulary, Matrix Reasoning, Block Design)

The State-Trait-Anxiety-Inventory (STAI) is a self-report instrument based on the differentiation between the presence and severity of temporary anxiety symptoms (State-Anxiety) and the longstanding generalized Trait-Anxiety as a stable personality trait [47, 56]. The STAI consist of two scales containing 20 items each with one scale addressing Trait- and another scale State-Anxiety.

The German version [46] of the Beck Depression Inventory-II (BDI-II) is a widely used self-report 21-item questionnaire for adolescents and adults from the age of 13 years. It is used to investigate the intensity of depression. Each item describes a particular symptom of depression with a list of four statements arranged in increasing severity. The responder has to choose one statement that best represents his/her feelings in the last two weeks [46]. Since the applicability of item 21 (loss of interest in sex) is limited for younger study participants, we excluded this item in our assessment.

Comorbidities will be assessed using the German Version of the Mini-international Neuropsychiatric Interview for Children and Adolescents (M.I.N.I.-KID 6.0) [44]. The M.I.N.I.-KID is a structured clinical diagnostic interview for DSM-IV and ICD-10 psychiatric disorders for children aged 6–17 years. As the version for adults, the M.I.N.I.-KID is organized in diagnostic sections (A–X). Each chapter contains Yes–No-questions grouped for each disorder in a language that is easy for children to understand [44]. Due to the high correlation between OXT release and the quality of attachment to parents and primary attachment figures, we measure attachment anxiety and avoidance with the German [57] short version of the Experiences in Close Relationships Scale-Revised Child version (ECR-RC) [58] for use in middle childhood and early adolescents. The ECR-RC is a self-report questionnaire on parent–child attachment containing two scales, each with 12 statements about the participant`s father or mother. Six statements rated from 1 (not at all) to 7 (very much) tap into attachment avoidance and 6 statements into attachment anxiety [58]. See Table 1 for an overview of the applied measures.

### Physiological measures and stress challenge

To minimize the effects of hormone fluctuations caused by the circadian rhythm [32], all participants will start the 2.5 h experimental test session (T2, see Fig. 1) at the same time in the afternoon (4:00 pm). Females who have begun menstruation will be tested in the luteal phase (time after ovulation) of their menstrual cycle, since the CORT stress response of females is more similar to the male reaction in the luteal phase than in the follicular phase [32, 59]. The onset of the female participants' ovulation and the pubertal development will be assessed using the German adaptation [60] of the self-report Pubertal Development Scale [61]. However, in previous studies, no main effect of sex for OXT levels in adolescents was found [32, 62]. Still, to minimize the effect of age and pubertal development [62, 63] both groups will be matched according to pubertal status [61].

At the end of T1, participants and their parents/legal guardians will be instructed to take one saliva sample by themselves at home within a relaxing atmosphere (T2a) and at least one hour after their last food intake. They will be asked to take the sample two days in advance of T2 in the afternoon, at the same time as the experimental test session (T2) takes place (4:00 pm). This will constitute the baseline salivary OXT and CORT level before the experimental test procedure (T2) will be conducted. The experiment will start with a 60 min relaxation period (T2a) to minimize external effects caused by previous food/drink intake, smoking cigarettes or stressful situations. During the experiment, participants will only be allowed to drink water [32]. Participants will be situated in a separate room (Room A) with relaxing material (reading, painting, puzzle, music). After 1 h (− 1 min before TSST start), the first salivary OXT and CORT level will be measured.

The TSST (T2b) will be conducted in room B in front of an unknown audience comprising two auditors and one experimenter (for more details see Kirschbaum et al. [33]). Participants will be introduced to the audience and informed about the upcoming task [42]. All participants have to give a free speech (5 min) for a mock job interview (application for a position as a student representative), for which he/she has 5 min to prepare. It will be feigned that this speech will be filmed and voice recorded [64]. Participants will be elucidated after the completion of the experiment. The second task will be an age-adapted mental arithmetic task (5 min), in which the participants will be asked to serially subtract a fixed number from a given starting number [42]. If the wrong number is calculated, the participant is asked to start the task from the beginning.

**Fig. 2:**
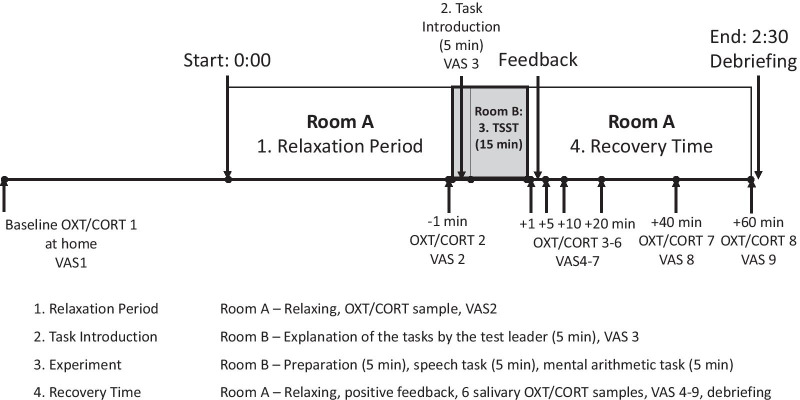
Overview of the test procedure. Timepoints of Measurement. OXT = oxytocin, CORT = cortisol, TSST = Trier Social Stress Test, VAS = Visual Analogue Scale, min = minutes

Upon completion of the TSST, participants will be seated in room A, thereby ending the stressful situation (T2c). To investigate the individual progression of coping with stress of each participant, no relaxing materials will be provided. Saliva samples will be taken at + 1, 5, 10, 20, 40 and 60 min after completing the tasks using Salivette^®^ (Sarstedt, Germany) collection devices and stored at − 20 °C until biochemical analysis. Salivary CORT will be measured at the laboratory of the Department of Biopsychology, Technical University of Dresden, Germany. Salivary OXT will be quantified by radioimmunoassay (RIAgnosis, Sinzing, Germany). In order to monitor the stress reaction during the procedure, heart rate will be recorded by an Ecg Sensor (EcgMove4, movisens, Germany) fixed with adhesive electrodes at the chest of each participant. Simultaneously to the physiological assessments, subjective levels of perceived stress, anxiety, insecurity and tension will be investigated using the Visual Analogue Scale (VAS) (Scale from 0 = feeling not stressed/anxious/insecure/tense at all to 100 = feeling highly stressed/anxious/insecure/tense, for more details see Hellhammer and Schubert, 2012 [37]) at 9 different time points [32]. In the relaxation period after the TSST, the experimenter will provide participants with positive feedback on their performance and the information that no video recording was made [32]. Finally, a clinical psychologist or doctor in charge will be available to talk in detail with the study participants and their legal guardians about the test procedure. He will also be available for advice/counseling if needed. See Fig. 2 for an overview of the test procedure.

### Power calculation

An a-priori power calculation was conducted using G*Power 3 [65] assuming a level of significance of *p *< 0.05, an effect size of f = 0.4 and two groups. The effect size of f = 0.4 was based on the study published by Bernhard et al. [32] and determined from the effect of TSST on CORT and OXT. The power analysis—when considering a correlation between CORT and OXT (as found by Bernhard et al. [32])—was conducted for a rmMANOVA (repeated measures Multivariate Analysis of Variance) and considered 8 measurements of CORT and OXT. Here, a sample size of 30 was estimated to achieve sufficient power of 80%. In case of no correlation between CORT and OXT, an rmANOVA (repeated measures Analysis of Variance) will be performed. Power analysis revealed that here a sample size of 8 will be required to achieve sufficient power of 80%. To determine the effects of group and sex on basal OXT and OXT increase through an additional MANOVA, a power analysis with an estimated effect size of f = 0.25 (medium sized effect) determined a total sample size of 64 (32 participants in each group). The latter sample size was chosen.

### Statistical analysis

To ensure objectivity the data will be assessed in accordance with the current standards of empirical research. The collected data will be evaluated using SPSS 25.0 (IBM Corp. Released 2017. IBM SPSS Statistics for Windows, Version 25.0. Armonk, NY: IBM Corp.). Using $x^{2}$—test for discrete analysis of variance (ANOVA) for continuous variables with group and gender as inter-subject factors, the demographic and psychometric data will be evaluated. The measured OXT and CORT levels will be transformed to achieve normal distribution. Correlations between psychological VAS ratings and OXT and CORT will be explored through Pearson correlations in case of normally distributed data and through Kendall’s τ otherwise. As correlation between OXT and CORT is expected, a rmMANOVA (repeated measures Multivariate Analysis of Variance) will be conducted to test the effects of time as a within-subject factor and group as a between-subject factor on OXT and CORT reactivity. In case of correlation between OXT, CORT and VAS ratings, VAS ratings will be included into the rmMANOVA, otherwise separate ANOVAs with the between-subject factor group and within-subject factor time will be conducted. In case of significant main effects or interactions, False Discovery Rate (FDR) [66] corrected post-hoc tests will be performed. Exploratory post-hoc analyses with FDR corrected p-values testing for effects of sex and age on OXT and CORT reactivity will also be conducted.

OXT and CORT increase will be calculated through area under the curve (AUC) analyses as described in Pruessner et al. [67]. Additionally, delta scores will be computed between the OXT and CORT baseline measure and the highest value for a measure of increase [68, 69] and between the highest OXT and CORT value and the lowest value after stress for a measure of recovery [70]. To determine group differences between OXT and CORT baseline, increase and recovery, MANOVAs will be computed with group as a between-subject factor and sex as a within-subject factor. In exploratory analyses, anxiety and depressive symptomatology scores will be included as covariates. As previous research has identified an effect of contraceptives on OXT baseline levels [38] a separate analysis will be run on female participants.

To assess the relationship between OXT and CORT reactivity, FDR-corrected correlations will be computed between CORT and OXT baseline levels and the increase and recovery measures. In case of significant correlations between CORT and OXT, a linear multiple regression model will be computed with CORT increase as dependent variable and OXT increase, CORT baseline and group as predictors to determine the amount of variance that is explained by OXT increase independently of baseline CORT. An additional regression model will be computed with CORT recovery as dependent variable and OXT increase and CORT increase as predictors. Effect sizes will be reported using Cohen’s *d* with 0.2 being considered a small, 0.5 a medium and 0.8 a large effect.

### Safety report

All participants will be informed in advance where to seek help for mental health problems during the procedure and that they can withdraw their consent for participating at any time. Because communication with strangers could induce increased stress levels, especially for anxious patients, participants may feel overburdened and symptoms of anxiety may be intensified during the stress test. If patients are unable to calm down through positive encouragement, the study will be terminated.

## Discussion

To our knowledge, the present study is the first controlled experimental observational study that investigates the endocrinological OXT and CORT reaction to an acute stressor in children and adolescents with anxiety disorders by assessing hormone concentrations in saliva. The only trial with healthy volunteers in this age-group was conducted by Bernhard et al. [32]. The aim of the present study is to contribute to a better understanding of the relationship between hormonal stress mechanisms and the etiopathogenesis of anxiety disorders. Despite the high prevalence of anxiety disorders in childhood and adolescence [1, 2] and the high comorbidity rate with other mental disorders [5], the neurobiological mechanisms of anxiety disorders in children and adolescents have rarely been investigated. It is of particular importance to identify neural biomarkers and neuromodulators that precede the onset of anxiety [71]. Early identification may support the improvement of preventive strategies and enhance cognitive, behavioral or pharmacological treatment options [15, 71]. Due to the standardized TSST protocol and the frequent saliva samples as well as the baseline sample, detailed information about the hormonal stress response will be collected over time. By measuring the heart rate continuously as well as the parallel measurement of CORT, any effects on the physiological stress response sparked by the implementation of the TSST will be measured. Although several experimental studies have established the stress-buffering and anxiolytic effect of OXT in rodents and adults [14, 15, 17], to our knowledge, the present study will be the first trial in human research that investigates whether there is a correlation between subjective stress-level, anxiety disorders and OXT release.

It is expected that participants suffering from anxiety disorders show a lower basal OXT level than healthy controls. If this is indeed the case, future studies may be able to achieve a therapeutic effect by raising basal OXT levels in clinical cohorts. As moderate anxiety and a lower basal OXT level correlated with higher OXT release during the TSST in healthy children and adolescents [32], the expected reduced OXT release of participants with anxiety disorders and a high level of anxiety may suggest the presence of a pathological lacking compensation mechanism. Since it has been shown that the OXT level peaks and decreases earlier than the CORT level [32], a present correlation between OXT level and CORT level suggest that OXT may influences the CORT release and subsequently the subjective stress response. Therefore, if less OXT is released, we expect a higher increase of CORT and respectively a higher subjective stress response. Alternatively, attenuated CORT release might support recent findings with the hypothesis of a CORT hypo-responsiveness in patients with anxiety disorder [39, 40, 42]. Identifying whether there is indeed such a relationship between OXT and CORT is crucial for the in-depth understanding of the stress response in children and adolescents suffering from anxiety disorders.

Potential limitations and challenges of the present study include the fact that the hormonal system is influenced by many external factors, such as pubertal status, sex and parent–child attachment. Therefore, a detailed inspection of the eligibility criteria (see Data Assessment) and a corresponding matching of the control group is highly important. All participants will be carefully examined with detailed questionnaires and categorical diagnoses will be assessed using a semistructured diagnostic interview in addition to routine clinical diagnostics.

For the healthy controls, the study provides the benefit of being screened by psychiatric professionals. If an unpredictable psychiatric abnormality is detected, the affected participant can be admitted directly to the in- or outpatient clinic of child and adolescent psychiatry, psychosomatics and psychotherapy at the University of Regensburg and hence receive help.

In summary, the present study is expected to make an important contribution to research on the endogenous stress reactivity in adolescents suffering from anxiety disorders and the significant role of OXT in the etiology of psychiatric disorders. A better understanding of the relationship between OXT and anxiety disorders may support future research on the therapeutic potential of OXT.

## Trial status

The recruitment of participants began in January 2020. The entire trial with the subsequent data assessment is scheduled to be completed by the end of 2021.

## Supplementary Information

**Additional file 1.** SPIRIT 2013 Checklist: Recommended items to address in a clinical trial protocol and related documents. The file contains the SPIRIT checklist according to the Standard Protocol Items: Recommendations for interventional Trials (SPIRIT) guidelines.

## Data Availability

The datasets used and/or analysed during the current study are available from the corresponding author on reasonable request.

## Abbreviations

ANOVA: Analysis of variance
rmANOVA: Repeated measures ANOVA
BAI: Beck anxiety inventory
BDI-II: Beck depression inventory-II
CORT: Cortisol
DSM-IV: Diagnostic and statistical manual of mental disorders-IV
ECR-RC: Short version of the experiences in close relationships scale—revised child version
HPA: Hypothalamic–pituitary–adrenal
ICD-10: International statistical classification of diseases and related health problems-10
i.n.: Intranasal
M.I.N.I-KID 6.0: Mini-international Neuropsychiatric Interview for children and adolescents 6.0
OXT: Oxytocin
OXTR: Oxytocin-receptors
PDS: Pubertal Development Scale
PTSD: Post-traumatic stress disorder
SCARED-D: Screen for Child Anxiety Related Emotional Disorders—German Version
SPAIK: Social Phobia and Anxiety Inventory for Children
SPIRIT: Standard Protocol Items: Recommendations for interventional Trials
STAI: State-Trait Anxiety Inventory
TSST: Trier Social Stress Test
VAS: Visual Analogue Scale for perceived stress, anxiety, insecurity and tension
WAIS-IV: Wechsler Adult Intelligence Scale—Fourth Edition
WISC-V: Wechsler Intelligence Scale for Children-V
